# Phase II trial of imatinib mesylate in patients with metastatic melanoma

**DOI:** 10.1038/sj.bjc.6604482

**Published:** 2008-08-19

**Authors:** K B Kim, O Eton, D W Davis, M L Frazier, D J McConkey, A H Diwan, N E Papadopoulos, A Y Bedikian, L H Camacho, M I Ross, J N Cormier, J E Gershenwald, J E Lee, P F Mansfield, L A Billings, C S Ng, C Charnsangavej, M Bar-Eli, M M Johnson, A J Murgo, V G Prieto

**Affiliations:** 1 Department of Melanoma Medical Oncology, The University of Texas M.D. Anderson Cancer Center, Houston, TX, USA; 2 ApoCell Inc., Houston, TX, USA; 3 Department of Pathology, The University of Texas M.D. Anderson Cancer Center, Houston, TX, USA; 4 Department of Cancer Biology, The University of Texas M.D. Anderson Cancer Center, Houston, TX, USA; 5 Department of Surgical Oncology, The University of Texas M.D. Anderson Cancer Center, Houston, TX, USA; 6 Department of Diagnostic Radiology, The University of Texas M.D. Anderson Cancer Center, Houston, TX, USA; 7 Department of Biostatistics and Applied Mathematics, The University of Texas M.D. Anderson Cancer Center, Houston, TX, USA; 8 The Cancer Therapy Evaluation Program, National Cancer Institute, Bethesda, MD, USA

**Keywords:** phase II, imatinib, metastatic melanoma, protein tyrosine kinases, antiangiogenesis

## Abstract

Metastatic melanoma cells express a number of protein tyrosine kinases (PTKs) that are considered to be targets for imatinib. We conducted a phase II trial of imatinib in patients with metastatic melanoma expressing at least one of these PTKs. Twenty-one patients whose tumours expressed at least one PTK (c-kit, platelet-derived growth factor receptors, c-abl, or abl-related gene) were treated with 400 mg of imatinib twice daily. One patient with metastatic acral lentiginous melanoma, containing the highest c-kit expression among all patients, had dramatic improvement on positron emission tomographic scan at 6 weeks and had a partial response lasting 12.8 months. The responder had a substantial increase in tumour and endothelial cell apoptosis at 2 weeks of treatment. Imatinib was fairly well tolerated: no patient required treatment discontinuation because of toxicity. Fatigue and oedema were the only grade 3 or 4 toxicities that occurred in more than 10% of the patients. Imatinib at the studied dose had minimal clinical efficacy as a single-agent therapy for metastatic melanoma. However, based on the characteristics of the responding tumour in our study, clinical activity of imatinib, specifically in patients with melanoma with certain c-kit aberrations, should be examined.

Imatinib (STI-571, Gleevec; Novartis Pharmaceuticals, Basel, Switzerland) is a small-molecule inhibitor of protein tyrosine kinases (PTKs), including the Bcr–Abl fusion protein, c-abl, abl-related gene (ARG), platelet-derived growth factor receptor (PDGFR)-*α*, PDGFR-*β*, and the c-kit tyrosine kinase receptor (Druker and Lydon, 2000; Schindler *et al*, 2000; Okuda *et al*, 2001). In cell-based assays, imatinib selectively inhibits PDGF- and stem cell factor-mediated cellular signalling, including ligand-stimulated receptor autophosphorylation, inositol phosphate formation, and mitogen-activated protein kinase activation and proliferation (Buchdunger *et al*, 1995, 1996). These properties suggest that in addition to treating chronic myelogenous leukaemia and gastrointestinal stromal tumours, imatinib may be useful in the treatment of diseases that involve abnormal activation of c-kit, PDGFRs, or other PTKs.

We previously demonstrated differential expression of PTKs in paraffin-embedded human metastatic melanoma specimens (Shen *et al*, 2003). Out of 31 specimens examined by immunohistochemistry (IHC), 17 (55%) expressed c-kit, 30 (97%) expressed PDGFR-*α*, 21 (68%) expressed PDGFR-*β*, 23 (74%) expressed ARG, and 27 (87%) expressed c-abl. These findings indicate that a subset of patients with metastatic melanoma may have tumours that express imatinib's target PTKs. To evaluate the clinical efficacy of imatinib in patients with metastatic melanoma that expressed these PTKs, we conducted a phase II study of imatinib. We performed tumour biopsies before and during treatment to examine genetic mutations and PTK expression and to assess the effects of imatinib on apoptosis of tumour and tumour-associated endothelial cells.

## Materials and methods

### Patient selection

This was an open-label, phase II clinical trial. The protocol for this study was approved by the Institutional Review Board of The University of Texas M.D. Anderson Cancer Center. All patients gave written informed consent before enrollment.

Eligible patients were required to have histologically confirmed melanoma with measurable metastases and no more than one prior systemic treatment, not counting adjuvant interferon-*α*. A pretreatment tumour biopsy must have had at least 25% of the tumour cells stain positive by IHC for PDGFR-*α*, PDGFR-*β*, c-kit (CD117), c-abl, or ARG for patients to enroll in this trial. Patients in whom the brain was the only site of metastasis or patients who had symptomatic central nervous system metastases were excluded. However, patients with small asymptomatic brain metastases were eligible.

### Treatment plan

Imatinib was administered orally at a dose of 400 mg twice a day until there was evidence of disease progression or unacceptable toxicity or until the patient refused to continue treatment. Because imatinib can irritate the gastrointestinal tract, patients were instructed to take the drug with meals. One course was defined as 6 weeks of treatment. All patients underwent tumour biopsies before treatment to assess target PTK expression by IHC. Patients were also asked to undergo a second biopsy during the second week of treatment for correlative studies.

### Response evaluation

In addition to routine radiological assessment including computed tomography at intervals of 6 weeks, patients' disease was also evaluated by positron emission tomography (PET) at baseline and after 6 weeks of treatment. Clinical responses were evaluated using the international criteria proposed by the Response Evaluation Criteria in Solid Tumors Committee (Therasse *et al*, 2000). Responses were evaluated after every 6-week course. Overall clinical response included both complete and partial responses. Time to progression, duration of response, and overall survival were measured from the start of treatment.

### Toxicity evaluation

Toxicities were evaluated and recorded according to the National Cancer Institute's Common Toxicity Criteria version 3.0. Patients were required to keep a daily record of adverse events and changes in concomitant medications. Complete blood counts and serum chemistry panels were obtained every 2 weeks.

### Receptor kinase expression

Immunohistochemical staining protocols were used to detect c-kit, c-abl, ARG, PDGFR-*α*, and PDGFR-*β* in paraffin-embedded tumour specimens (Shen *et al*, 2003). A rabbit antihuman antibody against c-kit (dilution, 1 ng ml^−1^) was purchased from DAKO (Carpinteria, CA, USA); the rabbit (PDGFR-*α*, PDGFR-*β*) and goat (c-abl) anti-human antibodies against PDGFR-*α*, PDGFR-*β* (dilutions, 1 ng ml^−1^), c-abl (dilutions 1 : 200), and ARG (dilutions 1 : 200) were from Santa Cruz Biotechnology (Santa Cruz, CA, USA). Antibody staining was optimised using tissue sections before antibodies were applied to tissue microarray sections. PDGFR-*α* and PDGFR-*β* were retrieved by treatment with pepsin at 37°C for 10 min, c-kit was retrieved by microwave treatment for 5 min, and no antigen retrieval was performed for c-abl or ARG. All primary antibodies were applied to the slides and incubated at 4°C overnight. Diaminobenzidine was used as a chromogen. Both the percentage of positive cells and the intensity of staining were graded by two dermatopathologists. The scoring system for IHC staining for the PTKs was assigned in the following way. For the percentage of cells staining positively for PTK: 0, 0–5%; 1, 6–25%; 2, 26–75%; 3, >75%; for the intensity of staining: 1, weak; 2, moderate; 3, strong.

### Tumour and endothelial cell apoptosis

Frozen tumour specimens were serially sectioned and stained for vessel count with an immunofluorescent antibody against the endothelial marker CD31. Apoptosis of tumour and endothelial cells was assessed by counting the number of cells (out of a total of 500) that stained positively with terminal deoxynucleotidyl transferase-mediated dUTP nick-end labelling (TUNEL). Sections were superimposed on one another by computer imaging to determine the degree of endothelial cell apoptosis at the invasive edges of the tumours.

### DNA sequencing

DNA was isolated from frozen tissue or from tissue stored in RNAlater (Ambion, Austin, TX, USA) using a QIAamp DNA minikit (Qiagen, Valencia, CA, USA) according to the manufacturer's instructions. RNA was extracted by standard methods using an RNeasy minicolumn (Qiagen) according to the manufacturer's instructions. cDNA was prepared using the two-step TaqMan reverse transcription reagent (Applied Biosystems, Foster City, CA, USA) according to the manufacturer's instructions, except that a primer specific for c-kit RNA (KIT 2961R, 5′-TTCCTGGAGGGGTGACCCAAACACT-3′) was used instead of random primers. The cDNA was then amplified by polymerase chain reaction. Nucleotide sequences were analysed with a 3730 × 1 DNA analyser (Applied Biosystems) at the M.D. Anderson Nucleic Acid Core Facility. The genomic DNA sequence of exons 9, 11, 13, 15, and, 17 was analysed as described previously (Chen *et al*, 2004).

### Statistical analysis

The statistical design of the study was based on a modified two-stage Simon's design (Simon, 1989). Twenty-one patients were to be enrolled and treated in the first stage of the study and 20 in the second stage, summing up to 41 patients. If the response rate was 5% (1 out of 21 patients) or less at the end of the first stage, the study would be terminated. If two or more patients responded, accrual would continue to a total of 41 patients. If the response rate at the end of the study was 10% (4 out of 41) or less, the regimen would be rejected for further study as a single agent for metastatic melanoma. If the response rate was higher, the drug would be recommended for further study. These calculations assumed there was 90% or greater probability that the regimen would be recommended for further study if the true efficacy rate was 20% or greater, and less than 5% probability if the true efficacy rate was 5% or less.

## Results

### Patient characteristics

From January 2002 to October 2003, 22 patients were enrolled and 21 were treated with imatinib. One patient with early progressive central nervous system metastases was not treated. The pretreatment characteristics of the patients are listed in Table 1. All 21 patients were evaluable for response and toxicity. Two patients (10%) had stage III melanoma with multiple unresectable in-transit lesions, and all others (90%) had stage IV melanoma.

All 22 patients enrolled had tumours expressing at least one target PTK. The baseline immunohistochemical data for the 21 treated patients who qualified the patients for enrollment are summarised in Table 2.

### Treatment

The 21 patients received a total of 33 courses of imatinib (median, one course per patient; range, 1–9). Three patients were unable to complete the first course of therapy because of rapid disease progression; one of them died of a progressing haemorrhagic brain metastasis. Thirteen and four patients completed one and two courses, respectively. The sole patient with responding disease tolerated nine consecutive courses of treatment over a period of 1 year. One patient with a history of rheumatoid arthritis required a dose reduction to 300 mg twice a day because of grade 3 polyarthritis. No other patient required a dose reduction.

### Responses

**Figure 1:**
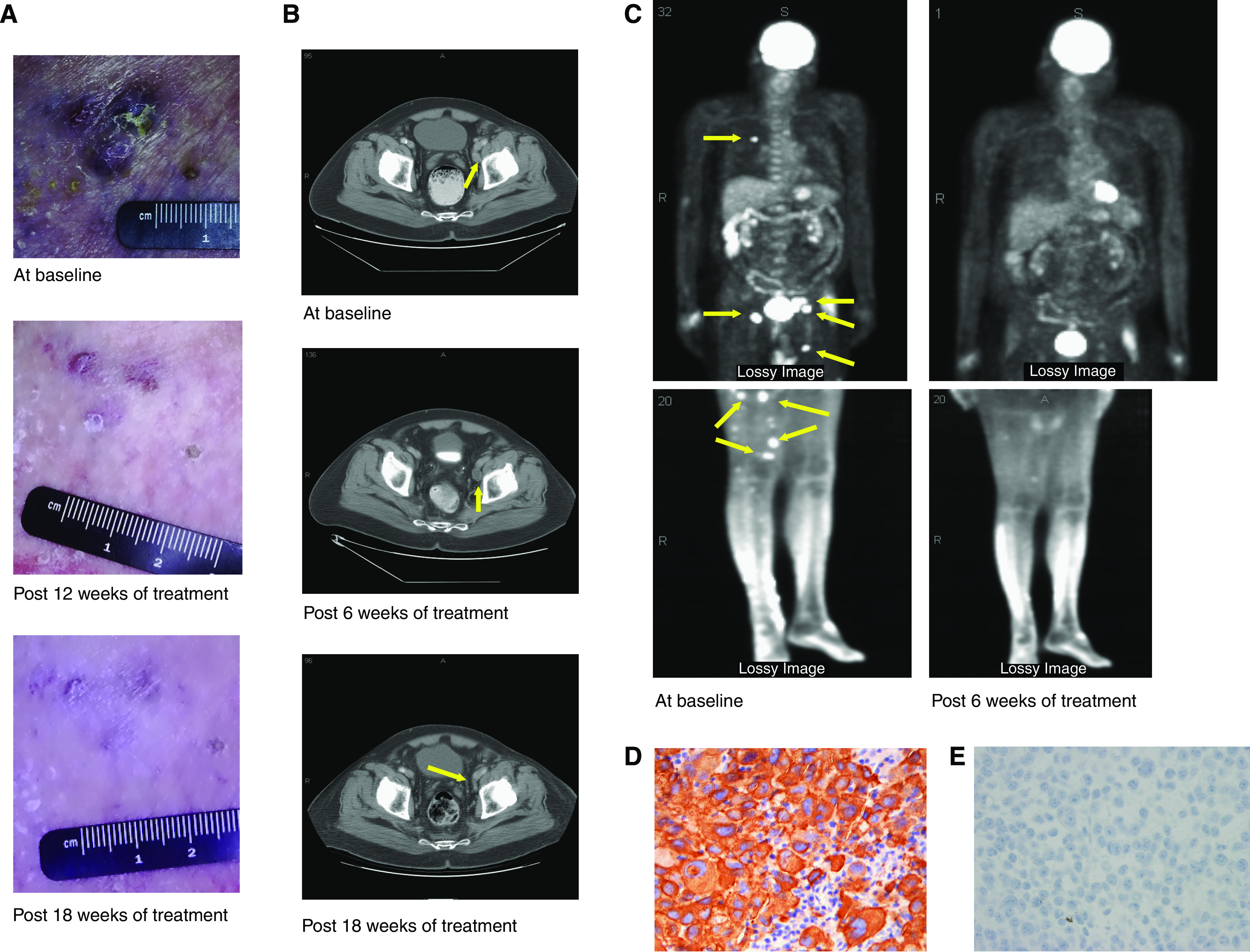
Clinical and radiological studies of a partial response to imatinib. All metastatic lesions shrank. (**A**) Response of in-transit metastases on right thigh. (**B**) Computed tomographic scan showing response of left external iliac lymph node (arrow). (**C**) Positron emission tomographic scans showing decrease in fluorodeoxyglucose uptake in all lesions (arrows). (**D**) Photomicrograph of the strong c-kit expression in the tumour of the responder. (**E**) Photomicrograph of a case of negative c-kit expression.

Clinical responses are listed in Table 2. Among the 21 patients treated, 4 had disease stabilisation, which lasted no more than 12 weeks. Only one patient (5%) had a partial response, which lasted for 12.8 months. That patient was a 66-year-old man whose disease had progressed while he had been receiving intratumoral HLA-B7 plasmid gene transfer treatment. He had a near-complete response to imatinib in numerous metastases of the cutaneous and subcutaneous tissues, inguinal and iliac lymph nodes, and lungs. After just 6 weeks of treatment, a PET scan revealed a marked reduction of metabolic activity in all disease sites (Figure 1). Follow-up computed tomography (CT) scans did not confirm ongoing major response in this patient until after 12 weeks of treatment. Serial physical exams with photographs confirmed gradual depigmentation and complete resolution of all palpable skin nodules and follow-up CT scans showed near-complete resolution in nodal and lung metastases after 6 months. The treatment was discontinued when he developed new brain metastases and a recurrence in the lymph nodes. This patient was later treated with temozolomide and paclitaxel achieving a brief partial response, but subsequently died of disease progression 26 months after the beginning of imatinib treatment.

With progression of disease confirmed in all patients, the median time to disease progression was 1.4 months (range, 0.6–12.8), and the median overall survival duration was 7.5 months (range, 0.7–25.8+), with two patients alive at 19 months.

### Toxicity

Adverse events are listed in Table 3 by toxicity grade and frequency. No patient required treatment discontinuation for toxicity. Fatigue and oedema were the only grade 3 or 4 adverse events that occurred in more than 10% of the patients, and these were reversible upon discontinuation of treatment.

### Correlative studies

We examined PTK expression in melanoma cells at baseline and during the second week of treatment (Table 2). Among the 21 patients treated, all with evaluable baseline tumour specimens, 16 also had specimens obtained during the second week of treatment. Thirteen of the second-week specimens were evaluable for PTK expression. With only one major response, no definite association could be made between the baseline immunohistochemical data and the observed clinical activity (Table 2A). Nevertheless, the patient with the major response had strong c-kit receptor staining in over 75% of the tumour cells – the strongest staining pattern observed among all the pretreatment specimens.

Among the evaluable 13 paired baseline and follow-up specimens, there were no consistent staining pattern changes observed after 2 weeks of treatment. Most often, however, there was a decrease in PTK receptor staining intensity (Table 2B). In the patient with the major response, c-kit receptor staining did not change, and there was complete loss of PDGFR-*β* and abl staining 2 weeks into treatment.

**Figure 2:**
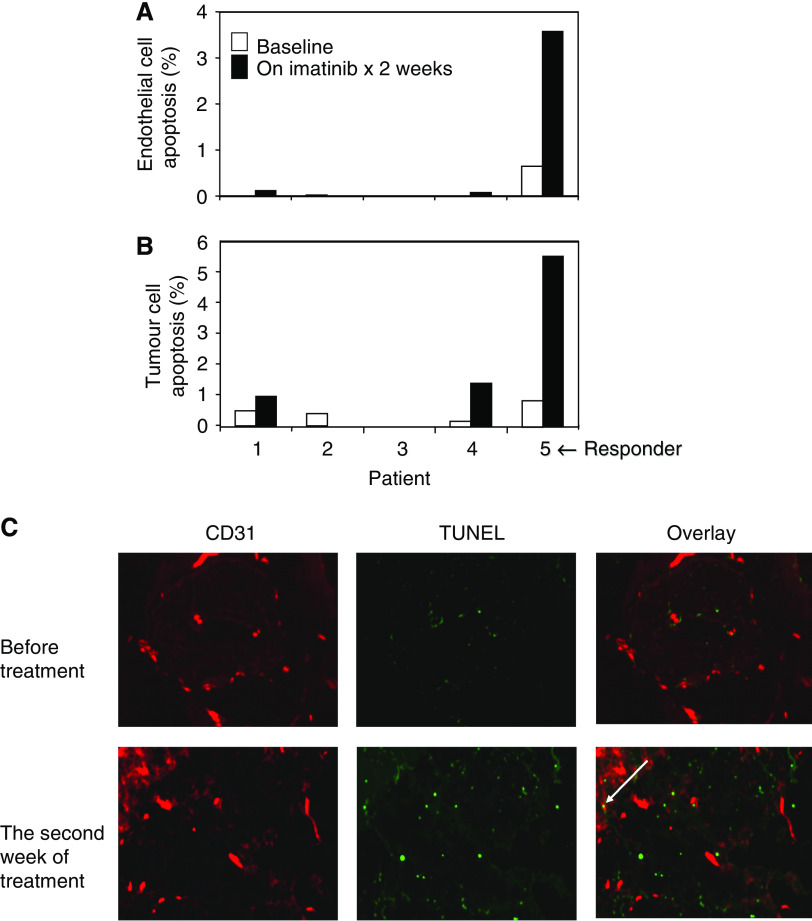
Tumour and endothelial cell apoptosis before treatment and during the second week of imatinib treatment. (**A**) Tumour cell apoptosis in five patients; patient 5 had a partial response. (**B**) Endothelial cell apoptosis in five patients. (**C**) Photomicrographs of the TUNEL stains for both melanoma and endothelial cells in the responder. Red in the CD31 column represents the endothelial cells, and green in the TUNEL column represents apoptotic cells. Yellow (indicated by an arrow) in the overlay column represents endothelial cells undergoing apoptosis.

The responding patient's tumours also showed an increase in the percentage of apoptotic endothelial and tumour cells after only 2 weeks of imatinib treatment, based on the CD31-TUNEL dual immunofluorescence studies, suggesting a very early impact of treatment on the tumour specimen (Figure 2).

**Figure 3:**
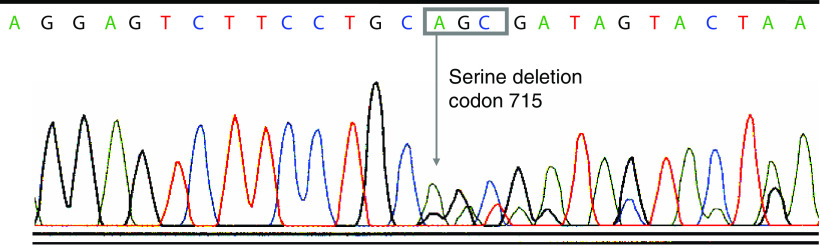
Chromatogram of the sequence for the alternative splicing seen in c-kit mRNA from the responding patient. The c-kit cDNA sequence from the responder displayed a deletion of the first three nucleotides of exon 15, which encoded a serine (codon 715).

We analysed pretreatment tumour samples from five patients, including the only responder, for c-kit mutation for both genomic DNA and cDNAs. None of our melanoma specimens analysed, including those from the responding patient, contained genomic mutations of c-kit in exon 9 or 11, which is frequently mutated in imatinib-responsive gastrointestinal stromal tumours (Heinrich *et al*, 2003). Genomic sequences of c-kit in exons 13, 15, and 17 were also analysed, and none of the specimens contained mutations in these sites. Of interest, the cDNA to RNA from the responder's tumour displayed alternative splicing in exon 15 of the c-kit kinase II domain (Figure 3). This alternate splice form had a 3 bp deletion of the codon for serine (codon 715) at the very beginning of exon 15. The alternate splice form encodes the SER^715−^ isoform of c-kit (Crosier *et al*, 1993; Lasota *et al*, 2002). However, this finding was not specific for the responding patient; four non-responding patients also had this alternative splice in their tumours.

There was no evidence of an immunological infiltrate in the responding metastases.

## Discussion

In this trial, imatinib was given orally at 400 mg twice a day without interruption. Wyman *et al* (2006) reported that imatinib administered at 400 mg twice a day caused unexpectedly severe toxicity with frequent grade 3 or 4 adverse events, requiring dose reduction and even treatment cessation. In another phase II study of imatinib, in which 18 patients with treatment-refractory advance melanoma were treated, Ugurel *et al* (2005) showed that there was no clinical response among 16 evaluable patients. However, in our study, 400 mg of imatinib given twice a day was fairly well tolerated. The toxicity of imatinib observed in our study was similar to previously reported clinical studies (van Oosterom *et al*, 2001; Verweij *et al*, 2003). Most adverse events, especially skin rash and pleural effusion, were self-limiting without drug discontinuation.

In our study, there was early progression of disease in the majority of patients and only one partial response, precluding the study from entering the second stage of accrual. This lack of efficacy was also observed in the other clinical trial testing the same dose and schedule of imatinib (Wyman *et al*, 2006). In that trial, the 26 patients enrolled with metastatic melanoma had a higher percentage of visceral metastases (stage M1c) and had received more prior systemic therapy compared with the patients enrolled in our study (58 *vs* 32% and 73 *vs* 27%, respectively). More grade 3 or 4 nausea, vomiting, fatigue, fluid retention with oedema, anaemia, hyponatraemia, and hypoglycaemia were observed in the second trial, perhaps as a result of these poorer prognostic characteristics. No clinical responses were reported. Hence, there has been only one partial response reported among the 46 evaluable patients treated in these two trials for an aggregate response rate of 2% and a median time to progression of less than 8 weeks.

Since all tumours screened in our study expressed at least one of the five target PTKs, based on positive immunohistochemical staining in at least 25% of the cells, the 25% threshold was not selective for predicting clinical response. Furthermore, there was only one clinical response among the 21 patients; therefore, positive IHC results, as defined in our study, were not predictive of response to imatinib. Nevertheless, in tumours from the one patient who had a meaningful and prolonged clinical response, over 75% of the cells had marked c-kit staining. None of the other patients' tumours had such intense staining; thus the threshold for positive staining, if used to select patients for treatment, could be set at a higher value in future studies. On the other hand, no change in the strong c-kit staining during the second week of treatment in the responding melanoma could be evidence that total expression of c-kit is not the critical marker for imatinib activity. Similarly, the decrease in staining intensity for PDGFR-*β* and c-abl in the responding tumour could be discounted as important leads as this pattern of staining was also seen in the non-responding specimens.

Despite the lack of correlative biological changes observed with imatinib in this patient, we believe that the lone response was not a coincidence because the response was observed in multiple sites including multiple in-transit lesions, deep inguinal nodes, and lung, and lasted more than a year on therapy. In addition, it is worth noting that the responder's primary disease was acral lentiginous melanoma. Recently, Curtin *et al* (2006) demonstrated that nearly 30–40% subtypes of melanoma, such as acral lentiginous, mucosal melanoma, or cutaneous melanoma, on chronic sun-damaged skin, harbour c-kit mutation and/or aberration, such as a gene amplification or an increased gene copy number. Although no genomic mutation in c-kit was found in the tumour of the responder in our trial, it is possible that the tumour contained either an increased c-kit gene copy number or its gene amplification, which might have activated the c-kit signal pathway. We found the alternative splice in exon 15, which encodes the SER^715−^ isoform of c-kit in tumour samples, but this variant, which has been reported in other tumour types by a number of investigators (Crosier *et al*, 1993; Ashman, 1999; Lasota *et al*, 2002), did not predict the response to imatinib.

The increase in apoptosis of both tumour and endothelial cells in the responding patient implies that imatinib may effectively inactivate important signal transduction pathways in endothelial cells as well. For instance, PDGFRs are known to be expressed both on melanoma and on endothelial cells (Beitz *et al*, 1991; Barnhill *et al*, 1996; Shen *et al*, 2003). The nature of the imatinib-sensitive signals that mediate survival of endothelial cells is being investigated. We are currently investigating the phosphorylation status of the target PTKs and the effect of imatinib on the inactivation of these targets in melanoma and endothelial cells of tumours from the patients in this study.

As reported in CML and GIST, resistance to imatinib was observed in the responder after 1 year of treatment. Many mechanisms have been suggested for this as well as ways to circumvent evolution of resistance in a previously exploitable pathway, including, but not limited to, substituting other PTK inhibitors or combining imatinib with other agents that affect other signal transduction steps. In addition, imatinib resistance might result from kinase-activating mutations of B-Raf, which is downstream of the target receptor kinases of imatinib. Davies *et al* (2002) recently showed that approximately 66% of melanomas contain somatic missense mutations, located within the kinase domain of B-Raf, with the ability to transform NIH3T3 cells. These mutations can constitutively activate the mitogen-activated protein kinase pathway, regardless of imatinib's ability to inactivate upstream receptor kinases. It may be necessary to select patients without such B-Raf mutations to increase the rate of clinical response to imatinib in patients with metastatic melanoma.

There is also increasing evidence that combinations of imatinib and chemotherapeutic agents, such as taxane or gemcitabine, have synergistic antitumour activity *in vitro* and *in vivo* (Pietras *et al*, 2002; Hwang *et al*, 2003; Mathew *et al*, 2004).

In conclusion, single-agent imatinib had only minimal clinical efficacy in patients with metastatic melanoma. The success of imatinib as a targeted therapy for advanced melanoma will depend on more stringent selection of patients with exploitable tumour targets and its use either sequentially or in combination with other agents. Currently, imatinib is under clinical investigation in patients with advanced melanoma harbouring somatic alterations of c-kit, and its result is eagerly anticipated.

## Figures and Tables

**Table 1:** Patient characteristics

**Characteristic**	**No. of patients (%)**
Total patients enrolled for the study	22
Total patients treated	21
Evaluable patients	21 (100)
	
*Sex*	
Male	13 (62)
Female	8 (38)
	
*Age (years)*	
Median	58
Range	33–83
	
*ECOG performance status*	
0	6 (29)
1	14 (67)
2	1 (5)
	
*Disease stage*	
III	2 (10)
IV	19 (90)
(M1a)	2 (10)
(M1b)	6 (29)
(M1c)	11 (52)
	
*Serum lactate dehydrogenase level*	
Normal	18 (86)
Higher than upper limit of normal	3 (14)
	
*Prior treatment*	
None	8 (38)
Interferon-*α* therapy (adjuvant)	8 (38)
Isolated limb perfusion	2 (10)
Biologic	2 (10)
Chemotherapy	4 (19)
	
*Pathologic type of primary melanoma*	
Superficial spreading melanoma	5 (24)
Nodular melanoma	2 (10)
Acral lentiginous melanoma	2 (10)
Melanoma of the soft part	1 (5)
Unknown primary	3 (14)
Unclassified or unspecified	8 (38)
	
*Site of primary melanoma*	
Upper extremities	3 (14)
Thumb – terminal phalanx	1 (5)
Lower extremities	9 (43)
Plantar surface	5 (24)
Scalp/face/neck	2 (10)
Torso	4 (19)
Unknown	3 (14)
	
*Site of metastases*	
Dermis/subcutaneous tissue	17 (81)
Lymph nodes	10 (48)
Lung	14 (67)
Liver	3 (14)
Bone	3 (14)
Brain	3 (14)
	
*Number of organs with metastases*	
Median	3
Range	1–5

ECOG=Eastern Cooperative Oncology Group.

**Table 2:** Receptor expression and clinical response

**Patient**	**c-kit**	**PDGFR-*α***	**PDGFR-*β***	**c-abl**	**ARG**	**Response**
*(A) Pretreatment*						
1	3/2	0/0	2/1	0/0	0/0	PD
**2**	**3/2**	**3/1**	**3/1**	**3/2**	**0/0**	**SD**
**3**	**0/0**	**0/0**	**3/1**	**2/1**	**0/0**	**SD**
4	3/2	0/0	0/0	0/0	1/1	PD
5	3/1	0/0	3/1	0/0	0/1	PD
**6**	**3/2**	**0/0**	**0/0**	**0/0**	**2/1**	**SD**
7	3/2	3/2	3/2	3/1	0/0	PD
**8**	**3/3**	**0/0**	**3/1**	**2/2**	**0/0**	**PR**
9	3/2	3/2	3/3	3/2	2/1	PD
10	2/1	2/1	0/0	2/1	0/0	PD
11	2/2	0/1	0/1	2/3	2/3	PD
12	1/1	0/1	1/1	1/1	2/2	PD
13	3/2	0/0	1/1	2/1	1/1	PD
14	3/1	3/1	3/1	3/3	3/2	PD
15	3/2	2/1	2/1	2/1	0/0	PD
16	3/1	0/1	0/1	1/1	0/0	PD
17	2/2	2/1	3/1	2/2	1/1	PD
18	3/2	3/2	3/2	3/2	3/2	PD
**19**	**2/1**	**3/3**	**2/1**	**1/1**	**0/0**	**SD**
20	2/1	2/1	2/1	0/0	1/1	PD
21	0/0	2/1	1/1	2/1	0/0	PD
						
**Patient**	**c-kit**	**PDGFR-*α***	**PDGFR-*β***	**c-abl**	**ARG**	**Response**
*(B) During second week of treatment*						
1	2/1↓	0/0**–**	0/0↓	2/1↑	0/0**–**	PD
**2**						**SD**
**3**	**0/0–**	**0/0–**	**0/0↓**	**2/2↑**	**2/1↑**	**SD**
4	3/2**–**	0/0**–**	0/0**–**	0/0**–**	0/0↓	PD
5						PD
**6**	**3/2–**	**0/0–**	**0/0–**	**2/1↑**	**2/1–**	**SD**
7						PD
**8**	**3/3–**	**0/0–**	**0/0↓**	**0/0↓**	**0/0–**	**PR**
	**2/2** ^*^	**0/0** ^*^	**0/0** ^*^	**0/0** ^*^	**0/0** ^*^	
9	0/0↓	1/1↓	1/1↓	1/1↓	0/0↓	PD
10	2/2↑	1/1↓	0/0**–**	2/1**–**	0/0**–**	PD
11	3/2↑	0/2↑	2/1↑	2/1↓	3/1**–**	PD
12	2/2↑	0/1**–**	3/2↑	0/0↓	1/1↓	PD
13	0/0↓	0/1↑	0/0↓	0/0↓	0/1↓	PD
14						PD
15						PD
16						PD
17						PD
18	1/1↓	0/0↓	0/0↓	0/1↓	0/1↓	PD
**19**	**0/0↓**	**0/0↓**	**0/0↓**	**0/0↓**	**0/0–**	**SD**
20						PD
21	1/1↑	1/2**–**	0/1↓	2/1**–**	0/0**–**	PD

ARG=abl-related gene; PD=progressive disease; PDGFR=platelet-derived growth factor receptor; PR=partial response; SD=stable disease.

The first number is an indicator of the percentage of cells staining positive for the protein tyrosine kinase (PTK) (0, 0–5%; 1, 6–25%; 2, 26–75%; 3, >75%); the second number denotes the staining intensity (1, weak; 2, moderate; 3, strong).

Blank spaces indicate no tissue available for analysis.

↑ denotes increase in protein expression during treatment.

↓ denotes decrease in protein expression during treatment.

**–** denotes no change in protein expression during treatment.

^*^ denotes PTK expression at the time of relapse (patient 8).

**Table 3:** Toxicities observed with imatinib treatment (number of patients with toxicity)

**Adverse event**	**Grade 1/2**	**Grade 3**	**Grade 4**
Fatigue	14	3	1
Anaemia	12	—	1
Oedema	13	3	—
Nausea	13	2	—
Muscle weakness	6	2	—
Anorexia	14	1	—
Lymphopoenia	10	1	—
Vomiting	9	1	—
Skin erythema/pruritis	9	1	—
Constipation	7	1	—
Dyspepsia	7	1	—
Neutropoenia	4	1	—
Ocular effects (visual changes)	8	—	—
Arthralgia	1	1	—
Dyspnoea	7	—	—
Diarrhoea	7	—	—
Pleural effusion	7		
Diaphoresis	6	—	—
Cough	6	—	—
Haemorrhage	6	—	—
Thrombocytopoenia	3	—	—
Fever	3	—	—
Serum creatinine elevation	3	—	—
Peripheral neuropathy	2	—	—
Petechia	2	—	—
Ascites	1	—	—

## References

[bib1] Ashman LK (1999) The biology of stem cell factor and its receptor C-kit. Int J Biochem Cell Biol 31: 1037–1051 10582338 10.1016/s1357-2725(99)00076-x

[bib2] Barnhill RL, Xiao M, Graves D, Antoniades HN (1996) Expression of platelet-derived growth factor (PDGF)-A, PDGF-B and the PDGF-*α* receptor, but not the PDGF-*β* receptor, in human malignant melanoma *in vivo*. Br J Dermatol 135: 898–904 8977709 10.1046/j.1365-2133.1996.d01-1092.x

[bib3] Beitz JG, Kim IS, Calabresi P, Frackelton Jr AR (1991) Human microvascular endothelial cells express receptors for platelet-derived growth factor. Proc Natl Acad Sci USA 88: 2021–2025 1848018 10.1073/pnas.88.5.2021 PMC51158

[bib4] Buchdunger E, Zimmermann J, Mett H, Meyer T, Muller M, Druker BJ, Lydon NB (1996) Inhibition of the Abl protein-tyrosine kinase *in vitro* and *in vivo* by a 2-phenylaminopyrimidine derivative. Cancer Res 56: 100–104 8548747

[bib5] Buchdunger E, Zimmermann J, Mett H, Meyer T, Muller M, Regenass U, Lydon NB (1995) Selective inhibition of the platelet-derived growth factor signal transduction pathway by a protein-tyrosine kinase inhibitor of the 2-phenylaminopyrimidine class. Proc Natl Acad Sci USA 92: 2558–2562 7708684 10.1073/pnas.92.7.2558 PMC42257

[bib6] Chen LL, Trent JC, Wu EF, Fuller GN, Ramdas L, Zhang W, Raymond AK, Prieto VG, Oyedeji CO, Hunt KK, Pollock RE, Feig BW, Hayes KJ, Choi H, Macapinlac HA, Hittelman W, Velasco MA, Patel S, Burgess MA, Benjamin RS, Frazier ML (2004) A missense mutation in KIT kinase domain 1 correlates with imatinib resistance in gastrointestinal stromal tumors. Cancer Res 64: 5913–5919 15342366 10.1158/0008-5472.CAN-04-0085

[bib7] Crosier PS, Ricciardi ST, Hall LR, Vitas MR, Clark SC, Crosier KE (1993) Expression of isoforms of the human receptor tyrosine kinase c-kit in leukemic cell lines and acute myeloid leukemia. Blood 82: 1151–1158 7688988

[bib8] Curtin JA, Busam K, Pinkel D, Bastian BC (2006) Somatic activation of KIT in distinct subtypes of melanoma. J Clin Oncol 24: 4340–4346 16908931 10.1200/JCO.2006.06.2984

[bib9] Davies H, Bignell GR, Cox C, Stephens P, Edkins S, Clegg S, Teague J, Woffendin H, Garnett MJ, Bottomley W, Davis N, Dicks E, Ewing R, Floyd Y, Gray K, Hall S, Hawes R, Hughes J, Kosmidou V, Menzies A, Mould C, Parker A, Stevens C, Watt S, Hooper S, Wilson R, Jayatilake H, Gusterson BA, Cooper C, Shipley J, Hargrave D, Pritchard-Jones K, Maitland N, Chenevix-Trench G, Riggins GJ, Bigner DD, Palmieri G, Cossu A, Flanagan A, Nicholson A, Ho JW, Leung SY, Yuen ST, Weber BL, Seigler HF, Darrow TL, Paterson H, Marais R, Marshall CJ, Wooster R, Stratton MR, Futreal PA (2002) Mutations of the BRAF gene in human cancer. Nature 417: 949–954 12068308 10.1038/nature00766

[bib10] Druker BJ, Lydon NB (2000) Lessons learned from the development of an abl tyrosine kinase inhibitor for chronic myelogenous leukemia. J Clin Invest 105: 3–7 10619854 10.1172/JCI9083 PMC382593

[bib11] Heinrich MC, Corless CL, Demetri GD, Blanke CD, von Mehren M, Joensuu H, McGreevey LS, Chen CJ, Van den Abbeele AD, Druker BJ, Kiese B, Eisenberg B, Roberts PJ, Singer S, Fletcher CD, Silberman S, Dimitrijevic S, Fletcher JA (2003) Kinase mutations and imatinib response in patients with metastatic gastrointestinal stromal tumor. J Clin Oncol 21: 4342–4349 14645423 10.1200/JCO.2003.04.190

[bib12] Hwang RF, Yokoi K, Bucana CD, Tsan R, Killion JJ, Evans DB, Fidler IJ (2003) Inhibition of platelet-derived growth factor receptor phosphorylation by STI571 (Gleevec) reduces growth and metastasis of human pancreatic carcinoma in an orthotopic nude mouse model. Clin Cancer Res 9: 6534–6544 14695158

[bib13] Lasota J, Kopczynski J, Majidi M, Miettinen M, Sarlomo-Rikala M (2002) Apparent KIT Ser(715) deletion in GIST mRNA is not detectable in genomic DNA and represents a previously known splice variant of KIT transcript. Am J Pathol 161: 739–741 12163399 10.1016/S0002-9440(10)64230-7 PMC1850714

[bib14] Mathew P, Fidler IJ, Logothetis CJ (2004) Combination docetaxel and platelet-derived growth factor receptor inhibition with imatinib mesylate in prostate cancer. Semin Oncol 31: 24–29 15176001 10.1053/j.seminoncol.2004.03.037

[bib15] Okuda K, Weisberg E, Gilliland DG, Griffin JD (2001) ARG tyrosine kinase activity is inhibited by STI571. Blood 97: 2440–2448 11290609 10.1182/blood.v97.8.2440

[bib16] Pietras K, Rubin K, Sjoblom T, Buchdunger E, Sjoquist M, Heldin CH, Ostman A (2002) Inhibition of PDGF receptor signaling in tumor stroma enhances antitumor effect of chemotherapy. Cancer Res 62: 5476–5484 12359756

[bib17] Schindler T, Bornmann W, Pellicena P, Miller WT, Clarkson B, Kuriyan J (2000) Structural mechanism for STI-571 inhibition of abelson tyrosine kinase. Science 289: 1938–1942 10988075 10.1126/science.289.5486.1938

[bib18] Shen SS, Zhang PS, Eton O, Prieto VG (2003) Analysis of protein tyrosine kinase expression in melanocytic lesions by tissue array. J Cutan Pathol 30: 539–547 14507401 10.1034/j.1600-0560.2003.00090.x

[bib19] Simon R (1989) Optimal two-stage designs for phase II clinical trials. Control Clin Trials 10: 1–10 2702835 10.1016/0197-2456(89)90015-9

[bib20] Therasse P, Arbuck SG, Eisenhauer EA, Wanders J, Kaplan RS, Rubinstein L, Verweij J, Van Glabbeke M, van Oosterom AT, Christian MC, Gwyther SG (2000) New guidelines to evaluate the response to treatment in solid tumors. European Organization for Research and Treatment of Cancer, National Cancer Institute of the United States, National Cancer Institute of Canada. J Natl Cancer Inst 92: 205–216 10655437 10.1093/jnci/92.3.205

[bib21] Ugurel S, Hildenbrand R, Zimpfer A, La Rosee P, Paschka P, Sucker A, Keikavoussi P, Becker JC, Rittgen W, Hochhaus A, Schadendorf D (2005) Lack of clinical efficacy of imatinib in metastatic melanoma. Br J Cancer 92: 1398–1405 15846297 10.1038/sj.bjc.6602529 PMC2362005

[bib22] van Oosterom AT, Judson I, Verweij J, Stroobants S, Donato di Paola E, Dimitrijevic S, Martens M, Webb A, Sciot R, Van Glabbeke M, Silberman S, Nielsen OS (2001) Safety and efficacy of imatinib (STI571) in metastatic gastrointestinal stromal tumours: a phase I study. Lancet 358: 1421–1423 11705489 10.1016/s0140-6736(01)06535-7

[bib23] Verweij J, van Oosterom A, Blay JY, Judson I, Rodenhuis S, van der Graaf W, Radford J, Le Cesne A, Hogendoorn PC, di Paola ED, Brown M, Nielsen OS (2003) Imatinib mesylate (STI-571 Glivec, Gleevec) is an active agent for gastrointestinal stromal tumours, but does not yield responses in other soft-tissue sarcomas that are unselected for a molecular target. Results from an EORTC Soft Tissue and Bone Sarcoma Group phase II study. Eur J Cancer 39: 2006–2011 12957454

[bib24] Wyman K, Atkins MB, Prieto V, Eton O, McDermott DF, Hubbard F, Byrnes C, Sanders K, Sosman JA (2006) Multicenter phase II trial of high-dose imatinib mesylate in metastatic melanoma: significant toxicity with no clinical efficacy. Cancer 106: 2005–2011 16565971 10.1002/cncr.21834

